# Fluorescence Microscopy Study of the Intracellular Sulfur Globule Protein SgpD in the Purple Sulfur Bacterium *Allochromatium vinosum*

**DOI:** 10.3390/microorganisms11071792

**Published:** 2023-07-12

**Authors:** Carolin Kümpel, Fabian Grein, Christiane Dahl

**Affiliations:** 1 Institut für Mikrobiologie & Biotechnologie, Rheinische Friedrich-Wilhelms-Universität Bonn, Meckenheimer Allee 168, D-53115 Bonn, Germany; ckuempel@uni-bonn.de; 2 Institut für Pharmazeutische Mikrobiologie, Rheinische Friedrich-Wilhelms-Universität Bonn, Meckenheimer Allee 16, D-53115 Bonn, Germany; grein@uni-bonn.de

**Keywords:** sulfur globules, sulfur oxidation, purple sulfur bacteria, *Allochromatium vinosum*, fluorescence microscopy

## Abstract

When oxidizing reduced sulfur compounds, the phototrophic sulfur bacterium *Allochromatium vinosum* forms spectacular sulfur globules as obligatory intracellular–but extracytoplasmic–intermediates. The globule envelope consists of three extremely hydrophobic proteins: SgpA and SgpB, which are very similar and can functionally replace each other, and SgpC which is involved in the expansion of the sulfur globules. The presence of a fourth protein, SgpD, was suggested by comparative transcriptomics and proteomics of purified sulfur globules. Here, we investigated the in vivo function of SgpD by coupling its carboxy-terminus to mCherry. This fluorescent protein requires oxygen for chromophore maturation, but we were able to use it in anaerobically growing *A. vinosum* provided the cells were exposed to oxygen for one hour prior to imaging. While mCherry lacking a signal peptide resulted in low fluorescence evenly distributed throughout the cell, fusion with SgpD carrying its original Sec-dependent signal peptide targeted mCherry to the periplasm and co-localized it exactly with the highly light-refractive sulfur deposits seen in sulfide-fed *A. vinosum* cells. Insertional inactivation of the *sgpD* gene showed that the protein is not essential for the formation and degradation of sulfur globules.

## 1. Introduction

A large proportion of sulfur-oxidizing bacteria form conspicuous sulfur deposits as intermediates during the oxidation of sulfide, polysulfides or thiosulfate [1,2,3,4]. The sulfur globules are deposited either extracellularly or intracellularly [1,5,6]. The formation of extracellular sulfur globules is characteristic of green sulfur bacteria and sulfur oxidizers of the gammaproteobacterial family *Ectothiorhodospiraceae*, while intracellular sulfur globules are typical of magnetotactic sulfur oxidizers, purple sulfur bacteria of the family *Chromatiaceae*, and sulfur-oxidizing bacterial endosymbionts [1]. Further spectacular organisms forming intracellular sulfur globules belong to the family *Thiotrichaceae*, which features some of the most conspicuous bacteria in nature. The species of the genera *Thiomargerita* and *Achromatium* as well as the filamentous sulfur-oxidizing bacteria of the genera, *Beggiatoa*, *Thiothrix* and *Thioploca* are among the largest known prokaryotes [7,8,9,10] and characterized by massive sulfur formation. Intracellular sulfur globules are spherical, highly light refractive and typically 1–3 μm in diameter, but sizes exceeding 15 μm have also been reported [11,12,13]. Our work indicated different speciation of the sulfur in the globules depending on the metabolic properties of the organisms and the environmental conditions: long sulfur chains possibly terminated by organic residues in purple sulfur bacteria, *cyclo*-octasulfur in chemotrophic sulfur oxidizers like *Beggiatoa alba* and *Thiomargararita namibiensis* and long chain polythionates in the aerobically grown acidophilic sulfur oxidizer *Acidithiobacillus ferrooxidans* [14,15]. It should be noted, however, that the chemical nature of the sulfur in the globules has been, and still is, the subject of intense controversy (reviewed in [1,16]). While sulfur globules appear to be randomly localised in many bacterial species, specific cellular localizations have also been reported. In *Thiovulum* for example, the globules accumulate toward one cell pole [17,18]. The general target compartment for intracellular sulfur storage is the bacterial periplasm, although the issue has not been definitively resolved in all cases [1]. In many organisms, including purple sulfur bacteria, *Thioalkalivibrio*, *Beggiatoa* and *Thiothrix* species, the intracellular sulfur globules are enveloped by 2–14 nm thick electron-dense layers consisting of one or more structural proteins similar to cytoskeletal keratins or plant cell wall proteins [1,19]. Genes encoding putative sulfur globule proteins targeted to the periplasm are present in almost all genome-sequenced, globule-forming Proteobacteria. The number of predicted *sgp* genes in a given organism can vary, e.g., there is only one in *Beggiatoa alba* and *Thiolinea disciformis*, but six in *Thiothrix caldifontis* [1].

A prominent and well-studied example of intracellular sulfur deposition are the sulfur globules of the anoxygenic phototrophic purple sulfur bacterium *Allochromatium vinosum* (class *Gammaproteobacteria*, family *Chromatiaceae*). In *A. vinosum*, sulfur globules reside in the periplasm, i.e., in the same cellular compartment containing periplasmic thiosulfate- and sulfide-oxidizing enzymes. This is evidenced by the presence of signal peptide coding sequences in the genes for the three structural proteins of the sulfur globule envelope that have been studied so far [5,20,21]. In *A. vinosum*, the so-called reverse-acting dissimilatory sulfite reductase (rDsr) system with rDsrAB as the key enzyme is essential for further oxidation of sulfur in the cytoplasm [22,23,24].

The sulfur globules of the *Chromatiaceae*, including those of *A. vinosum*, reach up to 1 μm in diameter [5] and account for up to 34% of the total cell dry weight [25,26]. The three established *A. vinosum* sulfur globule proteins SgpA (Alvin_1905, 13 kDa), SgpB (Alvin_0358, 12.6 kDa) and SgpC (Alvin_1325, 11 kDa) have in common that they are extremely hydrophobic. SgpA and SgpB are very similar in amino acid sequence and can functionally replace each other. SgpC participates in sulfur globule expansion [19,21]. In 2014, we re-evaluated the *A. vinosum* sulfur globule proteome and identified the protein Alvin_2515 as a new putative component, which we named SgpD [27]. The gene encoding Alvin_2515 lies next to genes for a hydrophobic/amphiphilic exporter and a probable two-component transcriptional regulator. Transcript levels for the *sgpD* gene dramatically increase on sulfide and thiosulfate compared to growth on malate in the absence of oxidizable sulfur compounds (28-fold and 6-fold, respectively) [28]. SgpD is synthesized with a cleavable Sec-dependent N-terminal signal peptide predicted to mediate transport to the periplasm. The protein could have a coil-coil structure typical of structural proteins, such as bacterial coiled-coil-rich cytoskeletal proteins, for example, crescentin from *Caulobacter crescentus* [27,29]. It should be noted, however, that a recent re-evaluation of coil-coil prediction tools revealed a high number of incorrect predictions, seriously questioning their informative value [30].

At present, direct experimental evidence for the function of SgpD as a sulfur globule protein is lacking. This would require definitive information on the intracellular localization of the protein and its possible association with sulfur deposits in vivo. Fluorescent reporter proteins, such as green fluorescent protein (GFP), are valuable non-invasive molecular tools for real-time in vivo imaging of living specimens and have the greatest potential to address these questions. One limitation of fluorescent proteins in pigmented phototrophic bacteria is signal quenching when they emit light at a wavelength absorbed by the pigments, as discussed for the anoxygenic phototrophic *Alphaprotebacteria Rhodobacter capsulatus* and *Rhodopseudomonas palustris* [31,32]. GFP is an example of a fluorescent protein that is incompatible with bacteriochlorophyll *a* and carotenoids, the pigments that drive photosynthesis not only in *R. capsulatus* and *R. palustris* but also in *A. vinosum*. In addition, commonly used variants of GFP are not suited for investigating the subcellular localization of periplasmic proteins. When exported to the periplasm in an unfolded conformation through the Sec system, they fail to fold properly and do not fluoresce [33]. However, very good alternatives to GFP, such as mCherry, mStrawberry, and tdTomato, derived from screening serial mutants, and genetically modified GFP [34,35,36], are now available and widely used for gene expression measurement, protein localization, in situ screening, and multi-omic profiling. Among them, the fluorescent protein mCherry stands out because of its bright signal, rapid maturation, high photostability, high N-terminal fusion tolerance, and excellent pH tolerance [37]. Importantly, red fluorescent protein derivatives such as mCherry do not share the transport difficulties of their GFP relatives and can be effectively transported through the Sec system [38,39].

Unfortunately, despite all its advantages, mCherry shares with almost all fluorescent proteins a strict requirement for molecular oxygen for the maturation of the fluorophore [40], making it inherently difficult to use under anaerobic conditions, such as during the phototrophic growth of *A. vinosum*. While there is no general limitation to aerobic systems, there is still not much known about the exact conditions that allow full maturation of the fluorophore after exposure of anaerobically grown cells to oxygen [41,42,43]. Flavin-based fluorescent protein presents an alternative, but all available derivatives have two major limitations. They produce only cyan-green fluorescence incompatible with the *A. vinosum* pigments and the fluorescence emitted is significantly dimmer compared to GFP [44] or newer anaerobic fluorescent reporters such as Fluorescence-Activating Absorption-Shifting Tag, FAST [45]. The latter has so far been used primarily in eukaryotic systems and, when used in bacteria, is best suited for secretion studies [44] and has only been applied under a limited number of conditions, e.g., in *E. coli* during nitrate or fumarate respiration [44,46]. SNAP-tag and Halo-Tag are further promising reporters for fluorescent labeling in the absence of oxygen, but so far have only been adapted to a few anaerobic bacteria, i.e., *Clostridium* species [47], which belong to the phylum *Bacillota*, and *Bacteroides thetaiotaomicron* (phylum *Bacteroidota*) [48].

After assessment of the available methods, we chose mCherry coupled to aerobic fluorescence recovery of the anaerobically produced protein as the most promising method for in situ fluorescence labeling and protein localization in the anoxygenic phototroph *A. vinosum*. Using mCherry we localized SgpD to the sulfur globules of the purple sulfur bacterium and collected evidence that its presence is not essential for sulfur globule formation.

## 2. Materials and Methods

### 2.1. Bacterial Strains, Plasmids, PCR Primers and Growth Conditions

The bacterial strains, plasmids and primers used in this study are listed in Table S1. All fluorescence experiments were carried out in a Δ*sgpD* strain derived from a spontaneous rifampicin-resistant mutant of the sequenced *A. vinosum* DSM 180^T^. *A. vinosum* was cultivated photoorganoheterotrophically in RCV medium [49] or photolithoautotrophically in Pfennig medium [50] without a reduced sulfur compound, referred to as “0” medium. Sulfide or thiosulfate was added as electron donors at the desired concentration. The cultivation was performed under anoxic conditions and continuous high illumination (about 2000 μE m^−2^ s^−1^) provided by incandescent light bulbs at 30 °C in completely filled screw-capped culture bottles or on agar plates. *Escherichia coli* was cultivated in Luria Bertani medium [51]. Antibiotics used for mutant selection were applied at the following concentrations (in µg mL^−1^): for *E. coli*: ampicillin (Roth) 100, kanamycin (Roth) 50, gentamycin (Serva) 25, for *A. vinosum*: rifampicin (AppliChem) 50, ampicillin (Roth) 10, kanamycin (Roth) 10, gentamycin (Serva) 2.

### 2.2. Recombinant DNA Techniques

Standard methods were used for molecular biological techniques. Chromosomal DNA of *A. vinosum* strains was obtained by a modified sarcosyl lysis [52]. The genotypes of the *A. vinosum* recombinants used in this study were confirmed by Southern hybridization or PCR. Southern hybridization was performed overnight at 68 °C. PCR amplification with *Taq* DNA polymerase and *Pfu* DNA polymerase were completed essentially as described previously [53]. DNA probes for Southern hybridization were digoxygenin labelled by PCR. Plasmid DNA from *E. coli* was purified using the GenJet^TM^ Plasmid Miniprep Kit (Thermo Scientific, Waltham, MA, USA).

### 2.3. Construction of Plasmids for Production of mCherry in A. vinosum

We wanted to clone mCherry under the strong *A. vinosum dsr* promoter and used the replicative plasmid pBBR1MCS2-L [54] as the basis. This plasmid contains an XbaI-HindIII fragment of the PCR-amplified *dsr* promoter fused to gene *dsrL* in XbaI-HindIII of pBBR2MCS-2. The plasmid was first supplied with a gentamycin resistance cartridge which had been amplified from pBRRMCS-5 with primers Gent-CpoI-Fw and Gent-CpoI-rev (Table S1). The amplicon was digested with RsrII and inserted into the RsrII site present within the kanamycin resistance cartridge of pBBRMCS-2, resulting in plasmid pBBR1-L-Gm. The mCherry gene was amplified via PCR with primers for-mCherry-NdeI and rev-mCherry-SalI with plasmid pmCherry [37] as the template. After digestion with NdeI/SalI, the amplicon was cloned between the SalI and NdeI sites of pBBR1-L-Gm, giving plasmid pBBR_dsr_mCherry_Gm that carries the gene for mCherry under the control of the *dsr* promoter. Fusion of mCherry with the potential signal peptide for SgpD and its first amino acid (tryptophan) as well as methionine was achieved by cloning a PCR product generated with primers for-mCher-sig and rev-mCherry-SalI with plasmid pmCherry [37] as the template between NdeI and SalI of pBBR1-L-Gm. The complete *sgpD* gene was amplified with primers fAlvin2515-NdeI and rAlvin2515-NdeI cloned into the NdeI site of pBBR_dsr_mCherry_Gm, resulting in a fusion of SgpD including its signal peptide and mCherry.

### 2.4. Fluorescence Microscopy

For microscopy, cells were transferred to a microscopy slide coated with a thin film of 1% (w/vol) agarose (Roth). Fluorescence microscopy was carried out at room temperature using a Zeiss Axio Observer Z1 microscope (Zeiss, Jena, Germany) equipped with an HXP 120 V light source (Zeiss, Jena, Germany), an αPlan-APOCHROMAT 100×/1.46 oil immersion objective (Zeiss, Jena, Germany) and an Axio Cam MR3 camera (Zeiss, Jena, Germany). Visualization of mCherry was achieved using Carl Zeiss filter set 64 HE (574–599 nm excitation, 605 nm beam splitter and 612–682 nm emission). Image acquisition and analysis were performed with Zen 2 software (Zeiss, Jena, Germany).

### 2.5. Construction of A. vinosum ΔsgpD::ΩKm

For the substitution of *sgpD* (Alvin_2515) in the genome of *A. vinosum* by a kanamycin cassette, SOE PCR [55] fragments were constructed using primer pairs Del-Alvin2515-fw1/Del-Alvin2515-rev1 and Del-Alvin2515-fw2/Del-Alvin2515-rev2 (Table S1). The resulting fragment was inserted into the mobilizable plasmid pSUP301 by HindIII restriction sites resulting in plasmid pSUP301_Δ*sgpD*. After digestion with EcoRI, the kanamycin cassette from pHP45ΩKm was ligated into the EcoRI site of pSUP301_Δ*sgpD*. The final mobilizable construct pSUP301_Δ*sgpD*_ ΩKm was transferred from *E. coli* S17.1 to *A. vinosum* Rif50 by conjugation [56]. Transconjugants were selected on RCV plates containing the appropriate antibiotics under anoxic conditions in the light. Double cross-over recombinants lost the vector-encoded ampicillin resistance.

### 2.6. Characterization of Phenotypes, Detection of Sulfur Species and Protein Determination

*A. vinosum* wild-type and the Δ*sgpD*_ΩKm strains were characterized in batch culture experiments essentially as described before [28]. Cells of *A. vinosum*, grown photoorganoheterotrophically on malate (RCV medium [49]) for three days was used as an inoculum for experiments concerned with the transformation of sulfide and thiosulfate. The culture volume of the precultures was 500 mL. Inoculum cells were harvested by centrifugation (10 min, 2680× *g*) and washed once in a “0” medium. The culture volume for phenotypic characterization was 250 mL. In these experiments, the starting optical density at 690 nm was set to 0.8–0.9. Sulfide, polysulfides, elemental sulfur and sulfate were either determined by HPLC [57] or using classical colorimetric or turbidometric methods as previously described [28,53].

## 3. Results

### 3.1. Suitability of mCherry for the Purple Sulfur Bacterium A. vinosum

**Figure 1:**
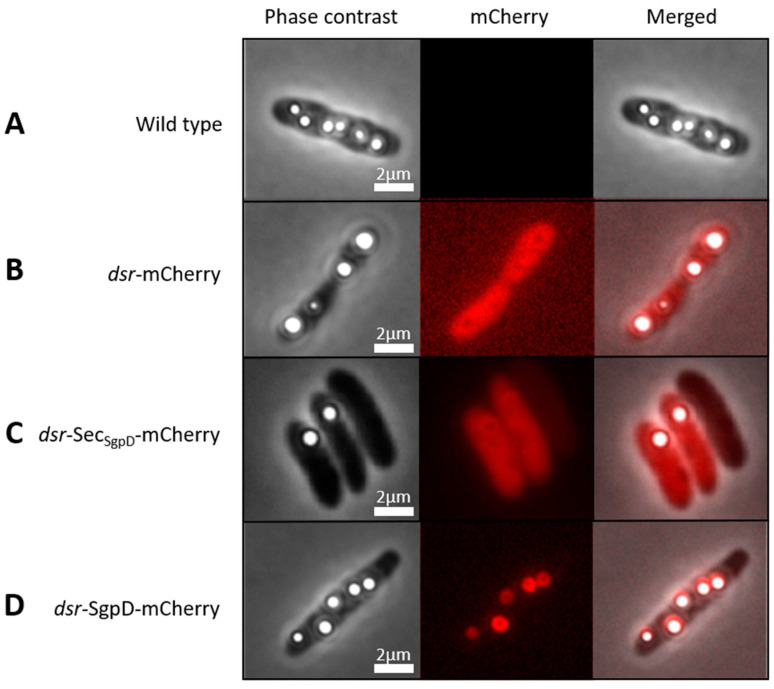
Phase contrast microscopy (left panels), fluorescence microscopy (middle panel) and merged phase contrast and fluorescence images of *A. vinosum* Rif50 wild type (**A**) and strains carrying pBBT_dsr_mCherry_Gm (**B**), pBBR_dsr_Sec_SgpD_, (**C**) or pBBR_dsr_SgpD_mCherry (**D**). Excitation times for phase contrast and the mCherry channel (587 nm) were 49.2 s and 1 s, respectively. All cultures were grown photolithoautotrophically in the light in the presence of 4 mM sulfide. Cells were collected by centrifugation when sulfur globule formation had set in and kept on ice until transfer to microscope slides on which the cells were exposed to air for 60 to 80 min before microscopy. Scale bar: 2 µm.

Here, we describe the production and validation of a direct fluorescence reporter for cell biological studies in an anoxygenic purple sulfur bacterium of the family *Chromatiaceae* (class *Gammaproteobacteria*). First, we show that intrinsic background fluorescence in the applied wavelength range is negligible (Figure 1A, middle panel). The next step was to determine whether mCherry is functional in *A. vinosum*, i.e., correctly expressed, translationally modified and folded. For this purpose, a reporter plasmid (pBBR_dsr_mCherry_Gm) was constructed in which the strong *A. vinosum dsr* promoter, which is inducible by sulfide, thiosulfate and elemental sulfur [28,54], was fused to the gene for the red fluorescent protein mCherry [37,58] targeted to the cytoplasm. The plasmid was transferred into *A. vinosum* via conjugation and the cells were then grown photolithoautotrophically in the light with 4 mM sulfide as an electron donor. Figure 1B shows that the cells were fully and homogeneously fluorescent after 70 to 80 min of exposure to air. Our results are fully consistent with experiments in the anoxygenic phototroph *R. palustris*, in which mCherry also became fluorescent when switched from anoxic to oxic conditions [32]. The spectroscopic properties of mCherry are the same irrespective of whether it is originally produced under aerobic or anaerobic conditions [32].

### 3.2. Fusions with mCherry Reveals Intracellular Localization of SgpD in A. vinosum

The putative sulfur globule protein SgpD is equipped with a probable Sec-dependent signal peptide proposed to direct its transport across the cytoplasmic membrane into the periplasm of the Gram-negative bacterium *A. vinosum* [27]. The predicted periplasmic localization of SgpD would have been a further difficulty when using the classical GFP chromophore which is known not to be correctly assembled in the bacterial periplasm [33]. However, this is not the case for mCherry and accordingly, brightly fluorescing cells were obtained when mCherry was fused to the signal peptide of SgpD (Figure 1C). *A. vinosum* is packed with intracytoplasmic membranes that are organized in vesicles (“chromatophores”). The interior of these chromatophores is extracytoplasmic, i.e., the entire cell body and not only its outermost layer as in other Gram-negative bacteria such as *E. coli* is filled with periplasmic space [20]. This fully explains the homogeneous distribution of mCherry when exported using the signal peptide (Figure 1C).

**Figure 2:**
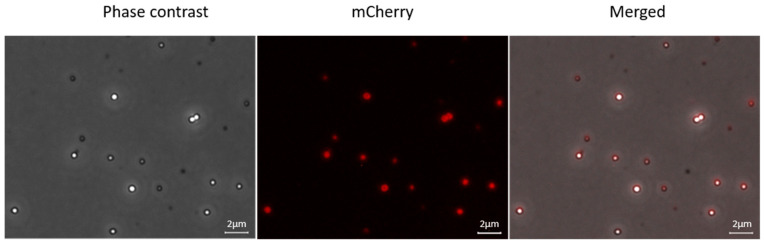
Phase contrast microscopy (**left panel**), fluorescence microscopy (**middle panel**) and merged phase contrast (**right panel**) and fluorescence images of sulfur globules isolated from *A. vinosum* Rif50 carrying pBBR_dsr_SgpD_mCherry. Excitation times for phase contrast and the mCherry channel (587 nm) were 49.2 s and 5 s, respectively. Scale bar: 2 µm.

In the next step, mCherry was fused to full-length SgpD including its signal peptide. Now, fluorescence was no longer evenly distributed in the cells but concentrated to circular structures inside the cells (Figure 1D, middle panel). Overlays of the fluorescence micrographs with images obtained by phase contrast microscopy showed that the circular structures exactly matched the position of sulfur globules in the cells and thus demonstrated that SgpD is attached to sulfur globules in vivo. Further evidence for this conclusion was obtained by microscopy of sulfur globules isolated from the *A. vinosum* strain with the SgpD-mCherry fusion (Figure 2). The sulfur globules were brightly fluorescent due to the attachment of mCherry via SgpD.

### 3.3. Stability of mCherry Fluorescence in A. vinosum

**Figure 3:**
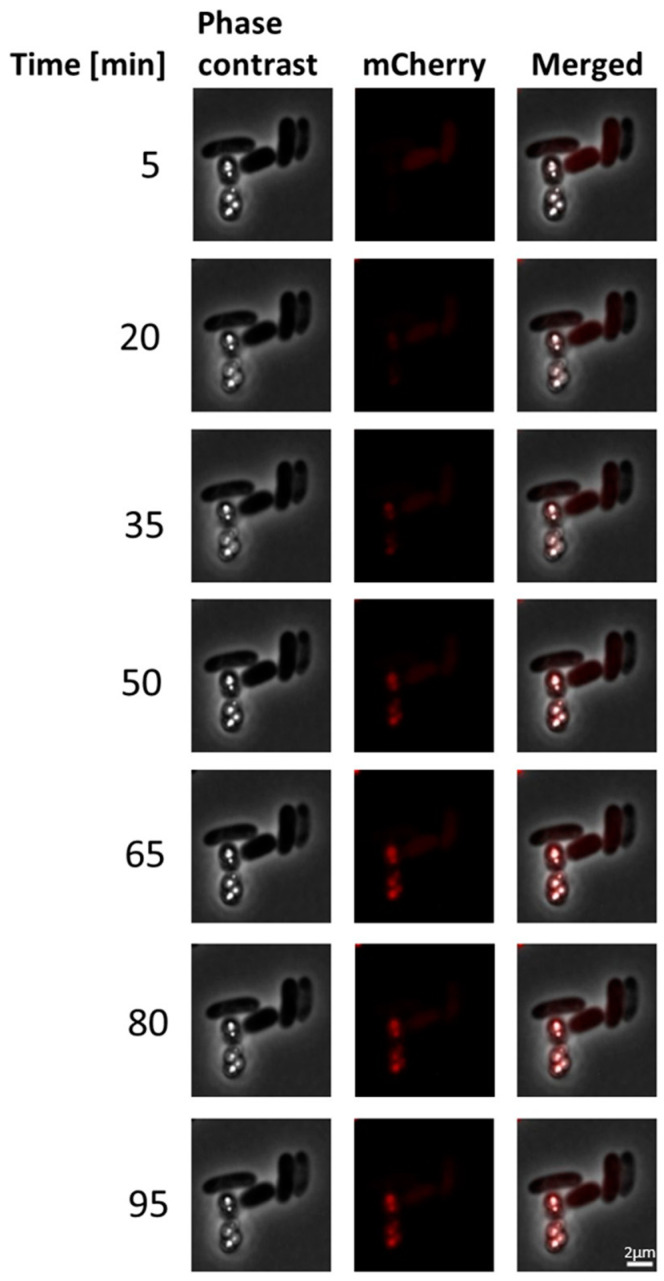
Development of fluorescence over time in *A. vinosum* Rif50 carrying pBBR_dsr_SgpD_mCherry. Left, middle and right panels show phase contrast microscopy, fluorescence microscopy and merged phase contrast and fluorescence images, respectively. Excitation times for phase contrast and the mCherry channel (587 nm) were 49.2 s and 1 s, respectively. Scale bar: 2 µm.

We sought to obtain more information about the development and stability of mCherry fluorescence and followed it via fluoresce microscopy over a period of 95 min (Figure 3). The fluorescence intensity gradually increased with oxygen exposure time and reached the maximum after a 60-min-exposure at room temperature. This maximum fluorescence was stably maintained for at least half an hour.

### 3.4. In Vivo Role of SgpD

The analysis of the *A. vinosum* sulfur globule proteome revealed SgpB as the second most abundant sulfur globule protein in this microorganism while SgpA and SgpC were less frequently detected [27]. The most abundant *A. vinosum* sulfur globule protein is SgpD [27]. The presence of a protein envelope around the sulfur inclusions in sulfur-oxidizing bacteria suggests an importantstructure–function relationship. Indeed, mutants of *A. vinosum* lacking SgpB and SgpC are no longer able to oxidize sulfide and thiosulfate and form sulfur inclusions [21]. SgpA and SgpB can replace each other in the presence of SgpC. The inactivation of *sgpC* resulted in the formation of much smaller sulfur globules suggesting that this structural protein plays a role in sulfur globule expansion [9]. Information about the in vivo role of SgpD has so far not been available and we therefore set out to fill this knowledge gap.

First, we re-evaluated SgpD distribution in bacteria and found that it is almost exclusively restricted to purple sulfur bacteria of the family *Chromatiaceae* with a threshold of e = 10^−5^ in Protein Blast searches. The only exceptions are a *Gammaproteobacteria* bacterium and a *Chromatiales* bacterium as well as the bacterial endosymbiont of *Escarpia laminata*, all of which cannot be strictly taxonomically grouped, but could also be members of the *Chromatiaceae*.

**Figure 4:**
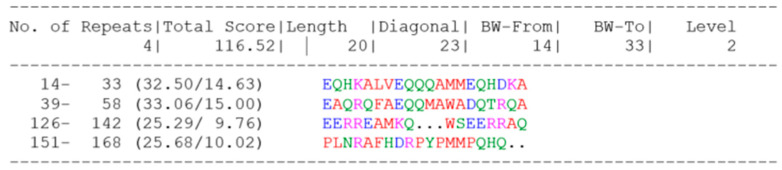
Alignment of repeats in SgpD without its signal peptide generated by RADAR. Notation: BW, best window; Level, depth of recursion; the alignment row reports first and last residue and, in parentheses, the alignment score and Z-score of the repeat. Red colors present small and hydrophobic, blue presents acidic, magenta indicates basic and green shows hydroxyl, sulfhydryl and amine residues.

With the other three sulfur globule proteins SgpA, SgpB and SgpC, *A. vinosum* SgpD shares a somewhat repetitive amino acid sequence with regularly spaced proline residues exemplified by the pattern P-X_2_-P-X_2_-P-X_4_-P-X_2_-P-X_2_-P-X_2_-P-X_5_-P-X_2_-PX_2_-P-X_2_-X_5_P-X_2_P in the central part of the protein molecule. Scanning for repetitive sequences by RADAR [59] revealed four repeats (Figure 4).

**Figure 5:**
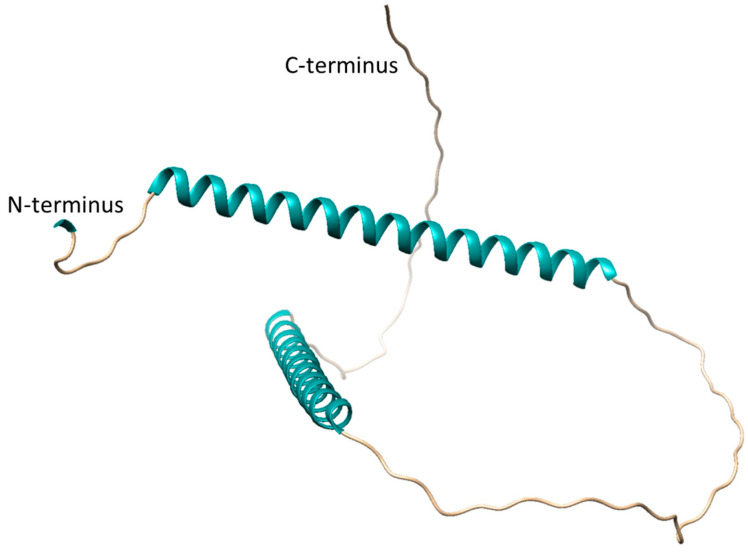
Alphafold [60] prediction of *A. vinosum* SgpD. The signal peptide (amino acids 1–22) that is cleaved off after transport to the cytoplasm is not shown.

An AlphaFold prediction for SgpD is available at https://alphafold.ebi.ac.uk/entry/D3RP35 (accessed on 1 June 2023) and is shown in Figure 5. Large predicted loop portions are modelled with low confidence (70 > pLDDT > 50). Two alpha-helices (amino acids 13 to 58 and 126 to 142) are predicted with very high confidence (pLDDT > 90). The first helix consists of repeats 1 and 2 shown in Figure 4, while the second helix is made up of the residues in repeat 3. Clearly, more experimental data is needed to gain further insight into the structural features of the proteins that make up the envelope of the sulfur globules.

In another approach to obtain information on the in vivo function of SgpD, we generated an *A. vinosum* Δ*sgpD* strain in which the gene was inactivated by the insertion of a kanamycin Ω interposon [61]. The nucleotide sequence between the ATG and the TAA stop codons of *sgpD* was completely replaced by a kanamycin Ω interposon. Just as the other *sgp* genes, *sgpD* is preceded by a promoter sequence predicted by BPROM [62] (TTGATA-N_13_-TTATATACT, ending 79 nucleotides upstream of the ATG start codon). An inverted repeat (CGGCCCCATCGACGATGGGGCCG) corresponding to the features of rho-independent transcription terminators [63] is found 45 nt downstream of the TAA stop codon. Thus, *sgpD* appears to form a separate transcription unit. Downstream polar effects of the Ω cassette should therefore be minimal. It should also be noted that the gene Alvin_2516, which follows *sgpD* at a distance of 216 nucleotides in the same direction of transcription, is under completely different transcriptional control, as its transcript abundance is not affected by the presence of sulfide or thiosulfate in the medium [28].

**Figure 6:**
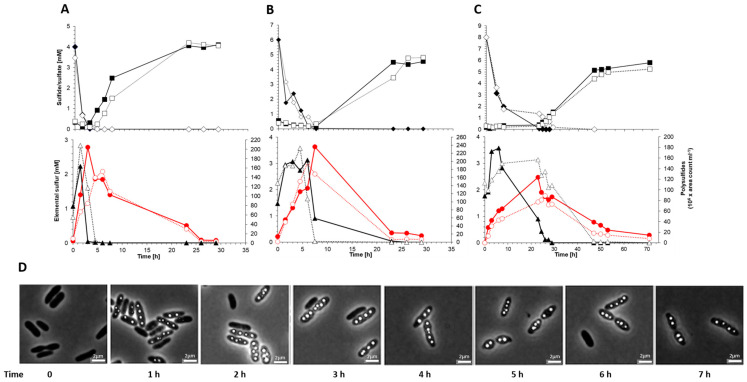
Consumption of 4 mM (**A**), 6 mM (**B**) and (8 mM) (**C**) sulfide by *A. vinosum* wildtype (filled symbols) compared to *A. vinosum* Δ*sgpD* (open symbols). Representative experiments are shown. The upper panels give sulfide (diamonds) and sulfate concentrations in culture supernatants. The lower panels show formation and degradation of the combination of the two main polysulfides (triangles) and intracellular sulfur (red circles). Part (**D**) shows phase contrast micrographs of *A. vinosum* Δ*sgpD* at the indicated time points after addition of 4 mM sulfide. Scale bar: 2 µm.

Photoorganoheterotrophic growth of the *sgpD*-deficient mutant strain on a malate-contianing medium with sulfate as the sole sulfur source was unaffected, thus excluding general growth defects. In addition, the ability of the mutant to grow photolithoautotrophically on sulfide-containing media and to form intracellular sulfur globules was not affected (Figure 6). Similar to the wild type, the mutant oxidized sulfide first to polysulfide and then to “zero-valent” polysulfane sulfur stored in sulfur globules, which was finally oxidized to sulfate (Figure 6A–C). The pattern of sulfur compounds in the growth experiments remained essentially the same regardless of the initial sulfide concentration of 2, 4 or 8 mM (Figure 6A–C). Lack of SgpD neither influenced the size nor the number of sulfur globules (Figure 6D). This result is similar to findings for SgpB and SgpA [5,21].

## 4. Discussion

The anoxygenic phototrophic bacterium *A. vinosum* is widely distributed in sulfide-rich, light-penetrated environments and serves as one of the very few genetically accessible model organisms of the purple sulfur bacteria [56,64,65]. Here, we have expanded the genetic toolbox for *A. vinosum* by establishing a basic cell biological method that now allows protein localization in the living cell using a fluorescent protein reporter, mCherry. We show that mCherry produced in *A. vinosum,* originally grown in the absence of oxygen under photolithoautotrophic conditions, is fully fluorescent within one hour of exposure of the cells to air. Interference from intrinsic background fluorescence is negligible. When exported to the periplasm, mCherry also develops full fluorescence and specifically accumulates at the cellular target of the protein to which it is fused. In the experiments reported here, mCherry linked to a potential protein of the proteinaceous sulfur globule envelope did indeed appear exclusively in the immediate vicinity of the intracellular sulfur deposits. To our knowledge, tagging with fluorescent proteins has so far not been established for any purple sulfur bacterium and we hope that the availability of the technique for *A. vinosum* will promote studies of the basic cell biology not only of this but also of other bacteria with the same physiology.

In the light, *A. vinosum* forms sulfur globules only under anaerobic conditions [20], while the red fluorescence protein mCherry needs molecular oxygen for the maturation of its chromophore. As pointed out by Jiang and coworkers [32], oxygen access into the fully folded barrel protein is of great importance, but may also trigger irreversible photobleaching and reduced photostability of a fluorescent protein [66]. In our experiments, the fluorescence signal of *A. vinosum* was fully recovered after exposure to oxygen for 60 min and was stably maintained at the maximum fluorescence intensity for at least 30 min. For comparison, fluorescence signals in the purple non-sulfur bacterium *R. palustris* required four hours of oxygen exposure for full recovery [32]. In any case, our results, as well as those obtained by others with *R. palustris*, show that mCherry produced under anaerobic conditions matures and develops fluorescence in the presence of air.

Coupling mCherry to SgpD allowed us to show that SgpD is tightly bound to sulfur globules and represents a novel component of their proteinaceous envelope, although it is neither essential for growth on sulfide nor for sulfur globule formation during its oxidation. Microorganisms such as *A. vinosum*, which live in sulfide-rich and oxygen-poor environments, appear to be equipped with an at least partially redundant set of proteins [1,21] that ensure the compatibility of massive sulfur deposition within the cell boundary with energy metabolism and the formation of new biomass. Having a variety of sulfur globule proteins that can compensate for each other to some extent may allow for fine-tuned adaptation to environmental conditions. Possession of SgpD may be advantageous at either low or relatively high sulfide concentrations that do not allow photolithotrophic growth of pure batch cultures due to insufficient electron supply or sulfide toxicity, respectively, but may be necessary or tolerated in an environment where other members of the community compete for the substrate or remove it rapidly enough to minimize toxic effects. Without question, a lot of further work is needed to elucidate the interaction of all sulfur globule proteins and to gain insights into the structure of these exciting proteins.

## Data Availability

Data are contained within the article and its supplementary material.

## Supplementary Materials

The following supporting information can be downloaded at: https://www.mdpi.com/article/10.3390/microorganisms11071792/s1, Table S1: Bacterial strains, primers and plasmids used in this study. References [37,54,61,67,68,69] are cited in the supplementary file.
