# Supplementary material for: Fluorescence Microscopy Study of the Intracellular Sulfur Globule Protein SgpD in the Purple Sulfur Bacterium Allochromatium vinosum
Source: Microorganisms. 2023 Jul 12;11(7):1792. doi: 10.3390/microorganisms11071792 (PMC10386293; doi:10.3390/microorganisms11071792)
Supplement: Supplementary file 1 [file microorganisms-11-01792-s001.zip › microorganisms-2486655-supplementary.pdf]

Table S1. Bacterial strains, primers and plasmids used in this study

Strain or Plasmid	Genotype or Phenotype	Reference
E. coli strains		
S17-1	294 (*recA thi pro* $hsdR^-M^+$) Tp^r Sm^r [RP4-2-Tc::Mu-Km:Tn7]	[67]
DH5*α*	F^- Φ80*lac*ZΔM15 Δ(*lac*ZYA-*arg*F) U169 *rec*A1 *end*A1 *hsd*R17 (rK–, mK+) *pho*A *sup*E44 $λ^-$ *thi*-1 *gyr*A96 *rel*A1	[68]
A. vinosum strains		
Rif50	Rif^r, spontaneous rifampicin-resistant mutant of DSM 180^T	[54]
sgpD:: Km	Rif^r, Km^r, *sgpD*:: Km (in *A. vinosum* Rif50)	This work
Plasmids		
pBBR1MCS-2	Km^r, Mob^+, *rep*, *lacZ*	[69]
pBBR1MCS-5	Gm^r, Mob^+, *rep*, *lacZ*	[69]
pBBR1MCS2-L	Km^r, XbaI–HindIII fragment of PCR-amplified *dsr* promoter and *dsrL* in XbaI–HindIII of pBBR1MCS-2	[54]
pBBR1-L-Gm	Gm^r, RsrII fragment of PCR-amplified gentamycin resistance cassette in RsrII of pBBR1MCS2-L	This work
pmCherry	Amp^r, mCherry	[37]
pBBR_dsr_mCherry_Gm	Gm^r, NdeI-SalI fragment of PCR-amplified gene for mCherry in NdeI-SalI of pBBR1-L-Gm, *dsr* promoter	This work
pBBR_dsr_Sec_{2515}_ mCherry_Gm	Gm^r, NdeI-SalI fragment of PCR-amplified gene for mCherry in NdeI-SalI of pBBR1-L-Gm, *dsr* promoter, PCR forward primer encoding *sgpD* signal sequence	This work
pBBR_dsr_sgpD_mCherry	Gm^r, NdeI fragment of PCR amplified *sgpD* (including signal peptide encoding sequence) in NdeI of pBBR_dsr_mCherry_Gm	This work
pHP45ΩKm	Ap^r, Km^r, pHP45Ω with kanamycin cassette	[61]
pSUP301	Ap^r, Km^r, RP4 oriT p15A ori Mob^+	[67]
pSUP301_Δ*sgpD*	Ap^r, Mob^+, HindIII fragment of PCR-amplified genome region around *sgpD* (Alvin_2515) with deletion of 603 bp of the *sgpD* sequence in HindIII of pSUP301	This work
pSUP301_Δ*sgpD*_ΩKm	Ap^r, Km^r, Mob^+, ΩKm cassette from pHP45ΩKm inserted into pSUP301_Δ*sgpD* using EcoRI	This work
Primers		This work
Del-Alvin2515-fw1	ATGATA**AAGCTT**CAACCTCGATTCGCCACCAC	This work
Del-Alvin2515-rev1	TGCGGCTTTTTA**GAATTC**CATGTGACACACCTTGAAAAGACAG (EcoRI)	This work
Del-Alvin2515-fw2	GTGTGTCACATG**GAATTC**TAAAAAGCCGCATGCGGGGTC (EcoRI)	This work
Del-Alvin2515-rev2	ATGATA**AAGCTT**CATCAGCCGGTCGTCGT	This work
Gent-CpoI-Fw	ATGATA**CGGTCCG**CTGTCGTGCCAGCTGCATTA (RsrII)	This work
Gent-CpoI-Rev	ATGATA**CGGACCG**ATCTCGGCTTGAACGAA (RsrII)	This work
for-mCherry-NdeI	ATAGCT**CATATG**GTGAGCAAGGGCGAGGA (NdeI)	This work
rev-mCherry-SalI	ATAGCT**GTCGAC**CCGCTACTTGTACAGCTCGTC (SalI)	This work
fAlvin2515-NdeI	ATAGCT**CATATG**TCCAAGCTGATCCAAACC (NdeI)	This work
rAlvin2515-NdeI	ATAGCT**CATATG**GCGCGTTTGGGAGGAAGG (NdeI)	This work
fo-mCher-SacIIV4	ATAGCT**CCGCGG**CGAACTGATTCGGGTCGAGCC (SacII)	This work
re-mCher-NdeIV4	ATAGCT**CATATG**GCGCGTTTGGGAGGAAGGC(NdeI)	This work
for-mCher-Sig	ATAGCT**CATATG**TCCAAGCTGATCCAAACCGTCTCCGCCATCG CGCTCGCTGCCGCCACGACCACGGCCTTCGCCTGGATGGTGAG CAAGGGCGAGGA (NdeI)	This work
re-Prom2515-NdeI	ATAGTC**CATATG**GTGACACACCTTGAAAAGACAG (NdeI)	This work

References

37. Shaner, N.C.; Campbell, R.E.; Steinbach, P.A.; Giepmans, B.N.; Palmer, A.E.; Tsien, R.Y. Improved monomeric red, orange and yellow fluorescent proteins derived from *Discosoma* sp. red fluorescent protein. *Nat. Biotechnol.* **2004**, *22*, 1567-1572.

54. Lübbe, Y.J.; Youn, H.S.; Timkovich, R.; Dahl, C. Siro(haem)amide in *Allochromatium vinosum* and relevance of DsrL and DsrN, a homolog of cobyrinic acid *a,c* diamide synthase for sulphur oxidation. *FEMS Microbiol. Lett.* **2006**, *261*, 194-202.

61. Fellay, R.; Frey, J.; Krisch, H.M. Interposon mutagenesis of soil and water bacteria: a family of DNA fragments designed for in vitro insertional mutagenesis of Gram-negative bacteria. *Gene* **1987**, *52*, 147-154

67. Simon, R.; Priefer, U.; Pühler, A. A broad host range mobilization system for in vivo genetic engineering: transposon mutagenesis in gram negative bacteria. *Nat. Biotechnol.* **1983**, *1*, 784-791.

68. Hanahan, D. Studies on transformation of *Escherichia coli* with plasmids. *J. Mol. Biol.* **1983**, *166*, 557-580.

69. Kovach, M.E.; Elzer, P.H.; Hill, D.S.; Robertson, G.T.; Farris, M.A.; Roop, R.M., II; Peterson, K.M. Four new derivatives of the broad-host-range cloning vector pBBR1MCS, carrying different antibiotic-resistance cassettes. *Gene* **1995**, *166*, 175-176.
